# Why pay more for robot in esophageal cancer surgery?

**DOI:** 10.1007/s13304-022-01351-0

**Published:** 2022-08-11

**Authors:** Fabrizio Rebecchi, Elettra Ugliono, Marco Ettore Allaix, Mario Morino

**Affiliations:** Department of Surgical Sciences, University of Turin, Torino, Italy

**Keywords:** Esophageal cancer, Esophagectomy, MIE, RAMIE, Costs

## Abstract

Esophagectomy is the gold standard for the treatment of resectable esophageal cancer. Traditionally, it is performed through a laparotomy and a thoracotomy, and is associated with high rates of postoperative complications and mortality. The advent of robotic surgery has represented a technological evolution in the field of esophageal cancer treatment. Robot-assisted Minimally Invasive Esophagectomy (RAMIE) has been progressively widely adopted following the first reports on the safety and feasibility of this procedure in 2004. The robotic approach has better short-term postoperative outcomes than open esophagectomy, without jeopardizing oncologic radicality. The results of the comparison between RAMIE and conventional minimally invasive esophagectomy are less conclusive. This article will focus on the role of RAMIE in the current clinical scenario with particular attention to its possible benefits and perspectives.

## Introduction

Esophagectomy is the mainstay of esophageal cancer treatment. However, esophagectomy is a highly invasive and challenging procedure that requires multiple accesses (abdominal, thoracic, cervical), and advanced surgical technical skills. In addition, it is burdened with prolonged operative time, significant morbidity and mortality. [1]

The surgical treatment of esophageal cancer has gone through an overwhelming transformation during the last two decades, with increasing application of minimally invasive techniques. Since the first description of thoracoscopic esophageal mobilization performed by Cuschieri in 1992, multiple minimally invasive approaches to esophageal cancer have been described, with a progressive shift from different hybrid procedures with a combination of minimally invasive and open approaches to a totally Minimally Invasive Esophagectomy (MIE) with a combined laparoscopic/thoracoscopic access. [2, 3] Since then, MIE has gained wide acceptance in most referral centers worldwide. [4, 5]

Randomized controlled trials, such as the TIME (Traditional Invasive versus Minimally Invasive Esophagectomy) trial and the MIRO (Oesphagectomie Pour Cancer par Voie Conventionnelle ou Coelio-Assisté) trial have demonstrated a significant reduction in postoperative complications and hospital stay, and better quality of life after both total minimally invasive and hybrid approaches compared to open procedures. [6, 7] Moreover, similar short- and long-term oncological outcomes were reported. [8]

MIE is a technically demanding procedure that requires advanced surgical skills mainly during the thoracoscopic phase, both in the mediastinal dissection and in the construction of the anastomosis. The technical challenges are mostly due to the narrow surgical space with limited compliance that does not allow the necessary mobility of the straight conventional thoracoscopic tools. This reflects into an increased risk of injury to the vital intrathoracic organs during the lymph node dissection and esophagogastric continuity restoration.

The application of robotic technologies to esophagectomy has been conceived to overcome the technical limitations of MIE. Robot-Assisted Minimally Invasive Esophagectomy (RAMIE) includes different surgical approaches: totally robotic (in which both the thoracoscopic and the abdominal laparoscopic phase are performed with the robotic assistance) or hybrid (a combination of robot-assisted thoracoscopy with a laparoscopic/open abdominal phase).

This review will critically analyze the outcomes of RAMIE, aiming at answering the question why we should pay more for this technology.

## Methods

We conducted a narrative review performing a comprehensive search of the literature on Pubmed and Medline databases, using a combination of the following terms: esophageal cancer, esophagectomy, minimally invasive esophagectomy (MIE), Robot-Assisted Minimally Invasive Esophagectomy, RAMIE, laparoscopic esophagectomy, open esophagectomy, learning curve, intrathoracic anastomosis, costs. We prioritized meta-analyses, systematic reviews and randomized controlled trials. Two authors (FR, EU) have selected the studies and extracted the data.

### Current literature evidence

#### RAMIE vs. open esophagectomy

To date, there is only one Randomized Controlled Trial (RCT) comparing RAMIE to open esophagectomy for cancer. The ROBOT trial was a superiority RCT designed to compare hybrid RAMIE (54 patients) and open esophagectomy (55 patients). All patients underwent a 3-stage transthoracic esophagectomy, and gastrointestinal continuity was restored with a cervical esophagogastric anastomosis. The robotic procedures were performed with a hybrid approach, consisting of a laparoscopic abdominal phase and a robot-assisted thoracoscopy. The primary endpoint was the rate of postoperative surgery-related complications. RAMIE was associated with significantly lower postoperative pulmonary, cardiac, and overall morbidity (63% vs. 80% RR 0.79 95% CI 0.62–1.00; *p* = 0.049), reduced postoperative pain, faster recovery, and better quality of life than open esophagectomy. The analysis of the intraoperative results showed that mean operative time was significantly longer in the RAMIE arm, while mean blood losses were significantly lower. No significant differences were observed in intraoperative complications (13 vs 16%). The conversion rate to open esophagectomy was 5%. Regarding the short-term oncologic outcomes, there were no differences between the two procedures in R0 resection rates and number of lymph nodes retrieved. [9] The results of the ROBOT trial have been confirmed by subsequent comparative studies, demonstrating that RAMIE has better early postoperative results, without compromising short-term oncological outcomes. [10–12] Table 1 summarizes the outcomes of studies comparing RAMIE and open esophagectomy published in the literature.

**Table 1:** Open esophagectomy vs. RAMIE: comparative studies

Authors	Year	Open (*N*)	RAMIE (*N*)	Operative time	R0 resection	Lymph nodes harvested	Overall complications	Respiratory complications
Sarkaria et al. [10]	2019	106	64	O < R	O = R	O <R	O = R	O > R
Gong et al. [11]	2020	77	91	O < R	O = R	O = R	O = R	O = R
Pointer et al. [12]	2020	222	222	O < R	O = R	N/A	O=R	O = R

*O* Open, *R* RAMIE, *N/A* not available

#### RAMIE vs. MIE

Three recent systematic reviews and meta-analyses comparing MIE and RAMIE have been published. [13–15]

For instance, Mederos et al. performed a meta-analysis of 9 propensity-matched studies comparing MIE and RAMIE and found fewer pulmonary complications with RAMIE but no statistically significant differences between the two procedures regarding overall complications, anastomotic leak, number of harvested lymph nodes, and mortality. [13] Similar results were reported by Angeramo et al., who performed a systemic review and meta-analysis of 60 studies comparing MIE (5275 patients) and RAMIE (974 patients) including only patients submitted to Ivor Lewis esophagectomy. In addition, the authors found that RAMIE was associated with higher rates of R0 resection (OR 2.84, 95%CI 1.53–5.26, *p* < 0.001) [14].

There is only one RCT: the RAMIE trial. [16] This multicenter RCT was designed to compare the outcomes of MIE (177 patients) and RAMIE (181 patients) in the treatment of esophageal squamous cell carcinoma. This study showed comparable results of the two approaches in terms of safety and feasibility, with similar rates of overall morbidity (48.6% vs. 41.8%, *p* = 0.19), pulmonary complications (13.8% vs. 14.7%, *p* = 0.82), anastomotic leakage (12.2 vs. 11.3%, *p* = 0.80), mortality (0.6% in both groups), and rate of R0 resections. RAMIE was associated with shorter operative time (203.8 vs. 244.9 min, *P* < 0.001) and a higher number of thoracic lymph nodes retrieved along the left recurrent laryngeal nerve (79.5 vs. 67.6%, *p* = 0.001).

The long-term results of the RAMIE trial and the results of other ongoing RCTs, the REVATE (Robotic-assisted Esophagectomy vs. Video-Assisted Thoracoscopic Esophagectomy) and the ROBOT-2 trials are awaited to fully elucidate the role of RAMIE over conventional MIE in the surgical treatment of esophageal cancer. [17, 18]

The results of the comparative studies are summarized in Table 2 [19–25].

**Table 2:** MIE vs. RAMIE: comparative studies

Authors	Year	MIE (*N*)	RAMIE (*N*)	Operative time	R0 resection	Lymph nodes harvested	Overall complications	Respiratory complication
He et al. [19]	2018	27	27	M < R	N/A	M = R	M = R	M = R
Chen et al. [20]	2019	54	54	M=R	M = R	M = R	M=R	M = R
Shirakawa et al. [21]	2020	51	51	M = R	N/A	M = R	M = R	M=R
Duanet al. [22]	2020	40	70	M = R	M = R	M < R	M = R	M = R
Gong et al. [11]	2020	144	91	M = R	M = R	M = R	M = R	M = R
Tsunoda et al. [23]	2020	45	45	M <R	M = R	M = R	M > R	M > R
Oshikiri et al. [24]	2021	51	51	M < R	N/A	M = R	M = R	M = R
Ninomiya et al. [25]	2021	30	30	M < R	M = R	M = R	N = R	N = R

*M* MIE, *R* RAMIE, *N/A* not available

### Why should we perform a RAMIE?

The current level of evidence does not demonstrate real clinically relevant advantages of RAMIE over MIE. However, possible reasons to prefer RAMIE rather than MIE may include advantages inherent to the robotic platform, the implementation of technical innovations, a shorter learning curve, and the cost reduction secondary to competitors entry in the market.

#### RAMIE: technical aspects

The technical characteristics of robotic technology include a magnified 3-dimensional (3-D) view of the operating field, the possibility to use articulated instruments with seven degrees of freedom, tremor filtering and motion scaling.

During RAMIE, the 3-D view and the use of articulated tools allow the surgeon to be more precise in the tissue dissection, thus leading to a more accurate lymphadenectomy and a lower risk of injury to surrounding organs. Tremor filtering and motion scaling let the surgical field to be stable over the entire procedure and, along with the 3-D view, allow to preserve small anatomical structures like the thoracic duct, the laryngeal nerve, to perform a more extended and precise lymphadenectomy and to suture in an easier and more accurate manner.

The benefits from these technical features are mostly evident during the thoracoscopic phase of RAMIE, where the limited and rigid intercostal space represents an anatomical obstacle to the movement of straight thoracoscopic tools. In addition, the enhanced freedom of movements gives the surgeon the possibility to choose among different types of anastomoses: mechanical (circular stapler vs linear stapler), hand-sewn and hybrid semi-mechanical. Even though the circular stapled anastomosis is the preferred anastomotic method during MIE, according to a recent survey of the Oesophago-Gastric Anastomosis Audit, the current evidence does not support one technique of anastomosis over another. [26] The rate of anastomotic leakage following Ivor-Lewis RAMIE is highly variable in the literature, ranging from 0 to 32%. [27–30] While no significant differences have been observed in anastomotic leak rate between the different types of anastomosis, a higher rate of anastomotic stricture is associated with circular stapled than hand-sewn anastomoses. [31–33] The anastomotic stenosis is responsible for long-term deterioration of quality of life and impaired nutritional status of the patients. One of the reasons why a stenosis less likely occurs in a semi-mechanical anastomosis is the wider dimension of the anastomosis. The use of the robotic platform, with its increased maneuverability and flexibility, may aid the construction of a semi-mechanical anastomosis which is technically demanding to be performed under conventional thoracoscopy. Further studies are needed to compare different anastomotic techniques in light of the possible technical advantages offered by the robotic technology.

The absence of tactile feedback and the lack of artificial intelligence are two major technical disadvantages of robotic technology. While the absence of tactile feedback can be compensated with adequate training and experience of the surgeon, the lack of the artificial intelligence does not currently allow the robotic system to interfere with the surgeon’s gesture. That means that the expertise of the surgeon, more than the robotic platform itself plays a crucial role to perform a good RAMIE. [34] Developments in artificial intelligence, along with digital connectivity and imaging integration in the next future are likely to add further benefits to the robotic system.

#### RAMIE: learning curve

The last two decades have witnessed a significant increase in surgical innovations aiming at improving patients healthcare. The use of some of these innovations requires a learning curve; this is particularly true for MIE and RAMIE. The MIE learning curve is in large part determined by the technical difficulties related to the thoracoscopic approach. The technical complexity of reflects into a long learning curve. [35, 36] The length of the learning curve of MIE ranges between 40 and 54 cases according to the operative time. [37] On the other hand the large multicentre study by van Workum et al., including 646 patients, considered the anastomotic leak rate as the primary outcome to define the learning curve. The length of the curve was 119 cases, with a significative decrease in leak rate from 18.8% of the initial phase to 4.5% after reaching the plateau (*p* < 0.001). Interestingly, 10% of patients that were operated during the learning curve had an anastomotic leakage. The authors speculated that the anastomotic leak might have been prevented if those patients had surgery performed by surgeons after completion of their learning curve. [38]

Recently, Kingma et al. presented the outcomes of 70 patients who underwent RAMIE performed by an experienced surgeon during the training pathway for the transition from MIE. The CUSUM analysis showed plateaus after 22 patients for operative time and intraoperative blood loss. Moreover, they observed a significant increase in the median number of lymph nodes harvested when comparing consecutive 23–70 patients to the first 1–22 cases (32 vs 23, *p* = 0.001). [39]

Similarly Hernandez et al. found a cut of 20 patients after that the intraoperative time significantly decreased. There are no clear data regarding learning curve and anastomotic leakage. [40]

Considering the complexity of RAMIE several structured training programs have been proposed. Proctorship is also widely used for a safe implementation of RAMIE to shorten the learning curve and improve the surgical outcomes. [24, 25]

#### RAMIE: economic considerations

The main obstacle to the diffusion of the robotic technology is related to costs. The robotic system requires a significant capital investment for its purchase and has substantial costs due to system maintenance and specific robotic semi-disposable instruments. Several studies have shown that the costs of the robotic approach to several abdominal diseases may be mitigated by reduce expenses related to postoperative complications and hospital stay. [41, 42] However, in the absence of specific studies comparing costs associated with RAMIE and MIE, it is unclear if it is the case also in esophageal cancer surgery. To achieve a better cost–benefit profile, and therefore to implement the utilization of the robotic system in esophageal surgery, it is essential to reduce expenses. Possible strategies to lower costs include optimization of operative room time utilization, increased number of “lives” of the robotic surgical instruments, lower costs of energy dissection tools and staplers. The establishment of a robotic program that involves different robotic teams (general surgeons, urologists, gynecologists, thoracic surgeon) may mitigate the initial costs of the robotic technologies by increasing the patients’ volume and by optimizing the interdisciplinary work [43] The entry of new competitors in the market will translate into lower pricing of the robotic equipment and speed up developments which is supposed to change the perspectives for RAMIE.

## Conclusions

MIE and RAMIE achieve similar early postoperative and oncological outcomes that are superior to those of open esophagectomy. The decision whether to perform RAMIE or MIE depends on surgeon preference and proficiency, and on economic availability of the surgical center. The augmented dexterity and accuracy offered by the robotic platform are the strengths to which a surgeon devoted to robotics hardly renounces in such challenging procedure.

In the near future, further reasons to perform RAMIE may come from the implementation of artificial intelligence, digital connectivity and imaging integration.

## Data Availability

All materials are available upon request.
